# Comparative analysis of dose calculation algorithms for CyberKnife-based stereotactic radiotherapy in lung cancer

**DOI:** 10.3389/fonc.2023.1215976

**Published:** 2023-10-02

**Authors:** Xuanchu Ge, Mingshan Yang, Tengxiang Li, Tonghai Liu, Xiangyu Gao, Qingtao Qiu, Yong Yin

**Affiliations:** ^1^ Department of Radiation Oncology and Physics, Shandong Cancer Hospital and Institute, Shandong First Medical University and Shandong Academy of Medical Sciences, Jinan, China; ^2^ Department of Urology, Shandong Cancer Hospital and Institute, Shandong First Medical University and Shandong Academy of Medical Sciences, Jinan, China; ^3^ Department of Radiation Oncology, Qilu Hospital of Shandong University, Cheeloo College of Medicine, Shandong University, Jinan, China

**Keywords:** CyberKnife, finite-size pencil beam, Monte Carlo, stereotactic body radiotherapy, non-small cell lung cancer

## Abstract

**Purpose:**

The accuracy of dose calculation is the prerequisite for CyberKnife (CK) to implement precise stereotactic body radiotherapy (SBRT). In this study, CK-MLC treatment planning for early-stage non-small cell lung cancer (NSCLC) were compared using finite-size pencil beam (FSPB) algorithm, FSPB with lateral scaling option (FSPB_LS) and Monte Carlo (MC) algorithms, respectively. We concentrated on the enhancement of accuracy with the FSPB_LS algorithm over the conventional FSPB algorithm and the dose consistency with the MC algorithm.

**Methods:**

In this study, 54 cases of NSCLC were subdivided into central lung cancer (CLC, n=26) and ultra-central lung cancer (UCLC, n=28). For each patient, we used the FSPB algorithm to generate a treatment plan. Then the dose was recalculated using FSPB_LS and MC dose algorithms based on the plans computed using the FSPB algorithm. The resultant plans were assessed by calculating the mean value of pertinent comparative parameters, including PTV prescription isodose, conformity index (CI), homogeneity index (HI), and dose-volume statistics of organs at risk (OARs).

**Results:**

In this study, most dose parameters of PTV and OARs demonstrated a trend of MC > FSPB_LS > FSPB. The FSPB_LS algorithm aligns better with the dose parameters of the target compared to the MC algorithm, which is particularly evident in UCLC. However, the FSPB algorithm significantly underestimated the does of the target. Regarding the OARs in CLC, differences in dose parameters were observed between FSPB and FSPB_LS for V_10_ of the contralateral lung, as well as between FSPB and MC for mean dose (D_mean_) of the contralateral lung and maximum dose (D_max_) of the aorta, exhibiting statistical differences. There were no statistically significant differences observed between FSPB_LS and MC for the OARs. However, the average dose deviation between FSPB_LS and MC algorithms for OARs ranged from 2.79% to 11.93%. No significant dose differences were observed among the three algorithms in UCLC.

**Conclusion:**

For CLC, the FSPB_LS algorithm exhibited good consistency with the MC algorithm in PTV and demonstrated a significant improvement in accuracy when compared to the traditional FSPB algorithm. However, the FSPB_LS algorithm and the MC algorithm showed a significant dose deviation in OARs of CLC. In the case of UCLC, FSPB_LS showed better consistency with the MC algorithm than observed in CLC. Notwithstanding, UCLC’s OARs were highly sensitive to radiation dose and could result in potentially serious adverse reactions. Consequently, it is advisable to use the MC algorithm for dose calculation in both CLC and UCLC, while the application of FSPB_LS algorithm should be carefully considered.

## Introduction

CyberKnife (CK) is a typical device used to implement Stereotactic body radiotherapy (SBRT). It generates highly conformal dose distributions around the Planning Target Volume (PTV) by using an accelerator mounted on a 6-axis robotic arm, achieving steep dose gradients at the PTV- organs at risk (OARs) boundary (1). The advantages of CK have been substantiated (2–5). Nevertheless, the complicated CK-based beams pose substantive challenges for dose computation, primarily in lung tumors due to the tissue’s heterogeneity between the high-density tumor and the neighboring low-density lung tissue (6). According to AAPM Report 85, even small dose differences can result in completely different treatment outcomes (7). Therefore, the accuracy of the dose algorithm in CK planning is vitally important for the treatment effect.

CK M6 system is outfitted with an InCise™ 2 multi-leaf collimator (MLC) while a finite size pencil beam (FSPB) algorithm has been specifically developed for it. However, the FSPB algorithm performs density correction only with regards to fluence and, thus, the accuracy of the calculated non-uniform area dose is subpar (8–10). The MLC-based Monte Carlo (MC) algorithm calculates the absorbed dose in tissue by statistically simulating physical processes based on the interaction of primary photon and secondary electrons in non-uniform region (11, 12). Typically, the MC algorithm is regarded as the gold standard for dose algorithms. Nevertheless, the dose optimization of the MC algorithm is computationally taxing, requiring more time to execute, especially for low levels of uncertainty (13). To tackle the drawbacks of FSPB and MC, Accuray incorporated lateral scaling correction, denoted FSPB_LS in this work, into the FSPB algorithm. Additionally, the FSPB_LS algorithm incorporates lateral scaling correction, including kernel density correction factors that contribute to the scattered radiation distribution along off-axis regions (10). A prior analysis conducted on a lung phantom revealed that almost all indicators showed no significant differences between MC and FSPB_LS algorithms. Since MC provides the tightest agreement with measurements, final dose calculations are suggested for inhomogeneous regions like the lung (14).

However, algorithmic dosimetric comparisons of radiotherapy planning, utilizing actual CT images of patients, have not yet been reported. Choosing an appropriate algorithm for dose calculation is a crucial step in the safe implementation of SBRT treatment in CK. Especially for central lung cancer (CLC) and ultra-central lung cancer (UCLC) in non-small cell lung cancer (NSCLC), the relationship between outcomes and safety has not yet been determined (15–18). Research indicates that patients with CLC who undergo SBRT treatment have documented cases of treatment-related deaths and a higher incidence of adverse events (17). For UCLC, researchers generally agree that UCLC is expected to carry a higher risk than other centrally located tumors. For example, even with a moderate dose, fatalities attributed to bronchial fistula have been documented in UCLC patients (19). Moreover, the implementation of SBRT for UCLC has been associated with the occurrence of various side effects such as trachea esophageal fistula, radiation pneumonitis, and pleural effusion (20, 21). The accuracy of dose calculation requirements for CLC and UCLC are markedly high, and due to their proximity to multiple OARs such as proximal bronchial tree (PBT), esophagus, aorta, and spinal cord, the complex structures present significant challenges to accuracy of dose calculation.

In this study, we employed three algorithms: FSPB, FSPB_LS, and MC, to calculate the doses in CK-MLC treatment plans for patients with CLC and UCLC. A comparison was made between the dose parameters in order to evaluate the potential of the FSPB and FSPB_LS algorithms as potential replacements for the MC algorithm, particularly the FSPB_LS algorithm.

## Materials and methods

### Patient characteristics

Upon obtaining approval from the Institutional Review Committee of Shandong Cancer Hospital and Institute, 54 patients with early-stage NSCLC who received SBRT treatment on TrueBeam (Varian Medical Systems, Palo Alto, CA) from 2017 to 2022 were enrolled in this study. This study selected NSCLC patients at stage IA-IB (tumor size ≤ 4 cm, N0M0), stage IIA (tumor size ≤ 5 cm, N0M0), or stage IB (tumor size > 5 cm and ≤ 7 cm, N0M0) as confirmed by histology. Staging criteria were in accordance with the 8th edition of the American Joint Committee on Cancer (AJCC). These patients were then classified into two categories, namely CLC and UCLC. CLC, in line with the recommendation of the International Association for the Study of Lung Cancer (IASLC), refers to a lesion located within 2 cm of isotropic expansion of all essential mediastinal structures like PBT, esophagus, heart, brachial plexus, aorta, spinal cord, phrenic and recurrent laryngeal nerve (22). For UCLC, the definition varies among authors (15, 16, 19, 20). Here, we defined UCLC as lesions with PTV adjacent/overlapping with PBT, aorta, or esophagus. The patient characteristics are shown in **Table 1**.

**Table 1:** Patient characteristics.

	CLC	UCLC
Number	26	28
Age	62 [34-77]	58 [39-72]
Male/Female	17/9	16/12
BMI	20.1 [17.6-24.3]	21.4 [17.2-23.5]
Volume of target (cm^3^)	21.06 [4.4 - 78.7]	22.45 [6.0- 44.2]
Clinic Stage I/II	15/11	19/9

### CT acquisition and target delineation

Each patient was scanned using a large aperture simulated CT scanner (Siemens Somatom Sensation, Munich, Germany). Ten phase-phase 4-dimensional CT (4DCT) images were acquired for each patient using a Real-time Position Management™ (RPM, Siemens Healthineers, Erlangen, Germany) system. The slice thickness of CT images was 3 mm. The 4D-CT images were transmitted to the Eclipse treatment planning system (TPS) (Varian Medical Systems, Palo Alto, CA) to reconstruct maximum intensity projection (MIP) and average intensity projection (AIP) images. Previous research indicates that fusing images from 10 phases into a MIP is a reliable clinical tool for generating the IGTV (internal gross tumor volume) from the 4DCT dataset. This significantly improves the efficiency of target delineation (23). However, compared to registration with AIP, registration with MIP significantly shifts the position of the patient bed downward. AIP is the preferred reference image for CBCT registration (24).

According to the RTOG 0236 protocol (25), the radiologist uses the lung window setting to contour the gross tumor volume (IGTV) on the MIP and expands the margin of the IGTV by 5 mm to generate the PTV, and radiation physicist use the image registration tool embedded in Eclipse to register the IGTV from MIP to AIP. All SBRT plans are designed based on the AIP image. Although KV planar images are used for registration in CyberKnife, considering that this study is simulation-based research and not for actual treatment, we still adopted the setting of using AIP images in this study. Ultimately, each patient’s AIP images were imported into Precision TPS for replanning.

### Treatment planning and dose prescription

We use gold fiducials tracking method to design the treatment plan for lung tumors, and virtually “place” 3 to 5 fiducial markers near the tumor. All plans were designed using multi-leaf collimators, no avoidance zones were set, and nodes for planning ranged from 26 to 54. All plans were initially calculated using the high-resolution model for the FSPB plan with a dose volume spacing of 0.98 mm×3mm×0.98 mm. For FSPB_LS, only the lateral scaling correction is added. Field direction, number of fields, number of nodes, and dose calculation resolution remain unchanged from the initial FSPB plan. The MC plans were calculated using the same high-resolution model with a clinically meaningful uncertainty level of 1%, this approach is based on the reference experience of previous similar studies (3, 26, 27), and other parameters remained unchanged from the initial FSPB plan. The CPUs used for dose calculations in this study are 2 processors of Intel Xeon E5-2620 v3 with 2.40GHz CPU Clock Speed

In the previous SBRT treatment, each patient was treated in 3-5 fractions, 7 to 11 Gy per fraction (Fx). In this study, we reset the prescription dose to 50Gy/5Fx. Additionally, maximum point normalization was employed during planning, requiring 100% of the prescribed dose to include at least 95% of the PTV and 100% of the prescribed dose to include 100% of the GTV. In planning for each algorithm utilized in the study, we have renormalized them in accordance with the aforementioned criteria.

### Evaluation of dosimetry

The dose statistics for FSPB, PSPB_LS and MC algorithm plans are based on dose volume histogram (DVH) analysis. We evaluated the following parameters of PTV: prescription isodose, target coverage, D_2_, D_5_, D_95_, D_98_ (the dose of 2%, 5% 95% and 98% volume of PTV), maximum dose (D_max_), mean dose (D_mean_), minimum dose (D_min_), conformity index (CI) and homogeneity index (HI). In this study, we used the CI recommended by Precision, whose expression is as follows:


(1) $$\text{CI}=\frac{\text{PIV}}{\text{TIV}}$$

where PIV = 3D volume of the prescribed isodose line, TIV = tumor volume covered by the prescribed dose. The closer the CI is to 1, it indicates that PIV and TIV completely overlap, and the conformity between the dose line and the target area is excellent.

The HI was defined as follows:


(2) $$\text{HI}=\frac{\text{Dmax(100\%)}}{\text{Rxdose}}$$

where D_max_ represents the maximum dose of PTV, and Rxdose is the prescribed dose of PTV. Ideally, 100% of the structures get 100% of the dose, i.e., (HI=1).

The following dosimetric parameters were selected for OARs evaluation: V_5_, V_10_, V_20_ and D_mean_ of the ipsilateral lung and contralateral lungs; V_30_, V_40_ and D_mean_ of the heart; D_max_ of the spinal cord, D_max_ and D_1cc_ of the ribs, D_max_ of the aorta, D_max_ and D_mean_ of the esophagus, and D_max_ of the PBT.

### Statistical analysis

The dosimetric parameters were compared with Wilcoxon rank-sum test or paired sample t-test, where it approtiate. P values were used to evaluate the difference and two-sided P<0.05 was considered statistically significant. All statistical calculations were implemented in Matlab software (version R2022a, The MathWorks Inc., Natick, MA 01760, USA) with statistics and machine learning toolbox.

## Result

### Dosimetric comparison of PTV and OARs in central lung cancer (CLC)

**Figure 1:**
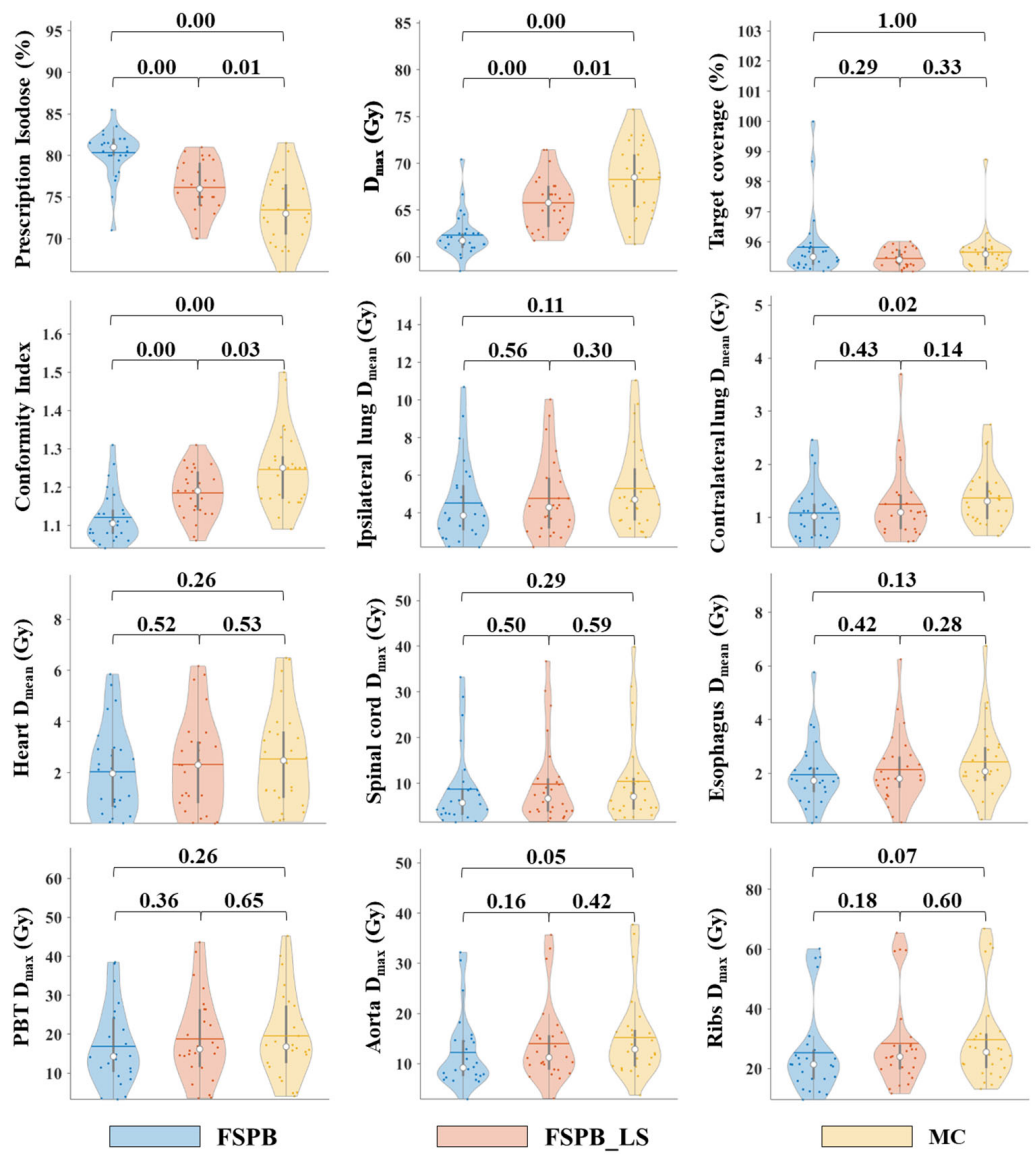
Violin plots of dose parameters for PTV and OARs in CLC, derived from FSPB, FSPB_LS, and MC algorithms. The horizontal lines within the violin plots represent the mean values, while the hollow circles depict the medians. The P-values are displayed above in the inset.

**Figure 2:**
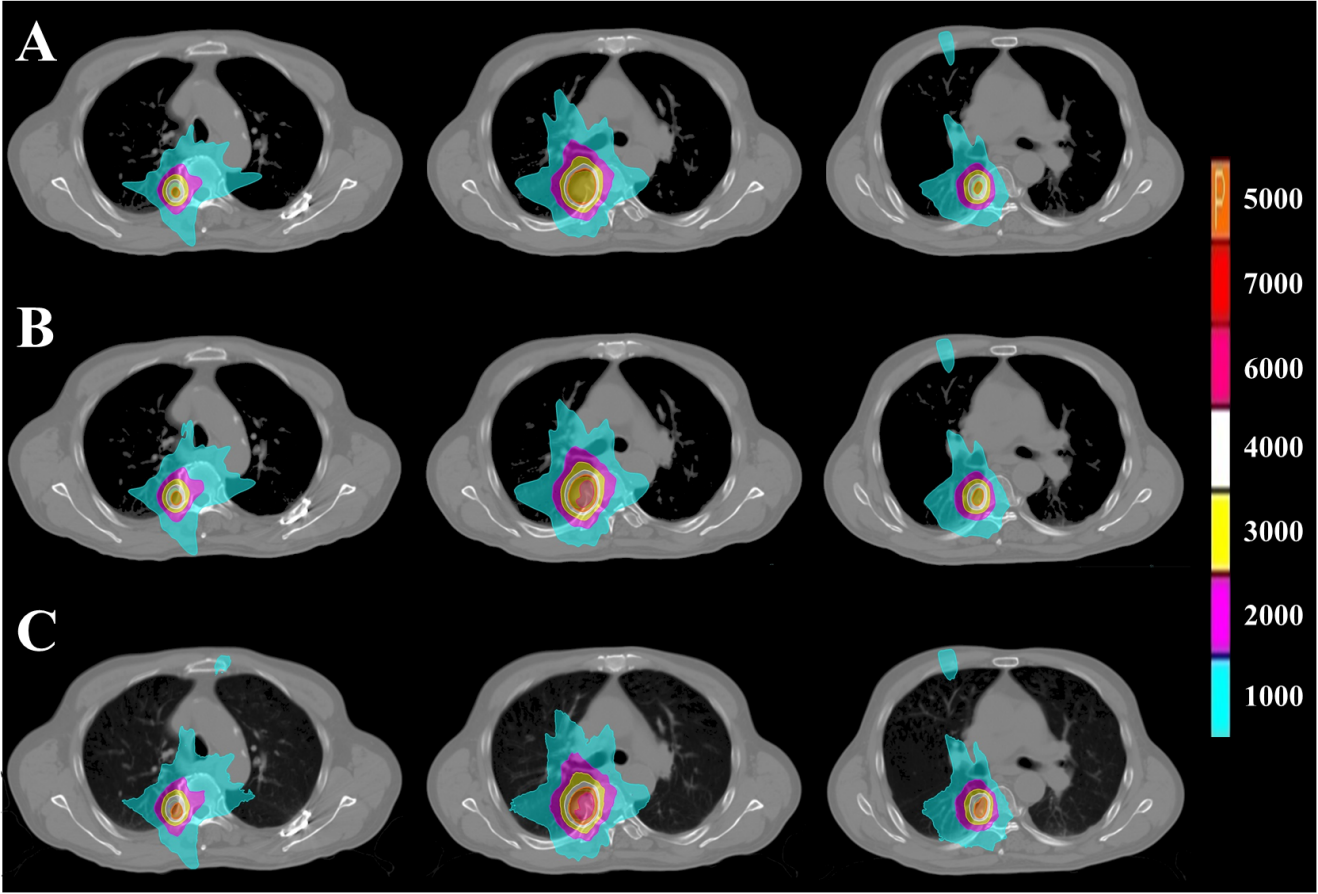
PTV dose distribution of representative patient with CLC based on FSPB, FSPB_LS and MC algorithms. The images from top to bottom illustrate **(A)** FSPB, **(B)** FSPB_LS, and **(C)** MC algorithms respectively. The images from left to right demonstrate the dose distribution of top, middle, and bottom layers of PTV along superior-inferior (S-I) direction. P represents the Prescription Dose.

**Figure 3:**
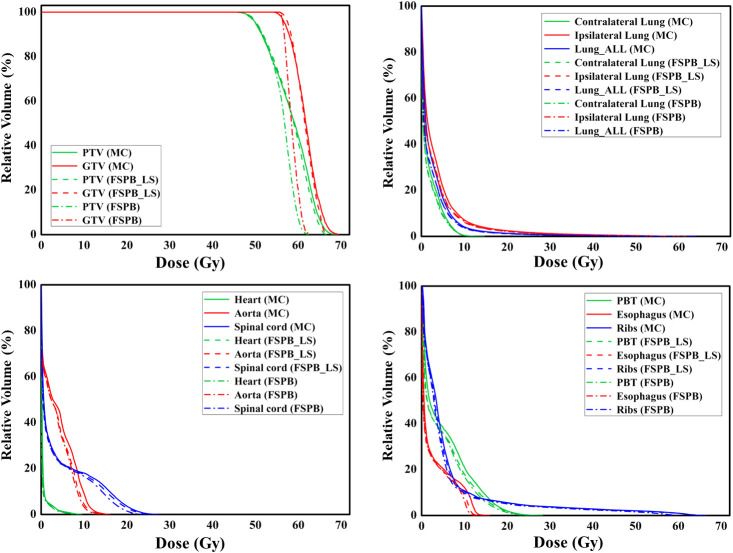
The dose-volume histograms (DVH) of PTV, GTV, and OARs planned through FSPB, FSPB_LS, and MC algorithms for the representative patient with CLC.

The dosimetric parameters of PTV were evaluated using the FSPB, FSPB_LS and MC algorithms, and the results are presented in **Table 2**. **Figure 1** shows the violin plots of the PTV and OAR dose parameters in CLC. The dose distribution and DVH for representative patient for all three algorithms were displayed in **Figures 2**, **3**, respectively.

**Table 2:** Dose-volumetric parameters of the PTV for CLC.

	FSPB	FSPB_LS	MC	P < 0.05
D_2_ (Gy)	61.29 ± 1.95	64.67 ± 2.40	65.99 ±3.39	a, b
D_5_ (Gy)	60.67 ± 1.81	63.84 ± 2.24	65.19 ±3.84	a, b
D_95_ (Gy)	50.34 ± 0.67	50.18 ± 0.11	50.26 ±0.34	
D_98_ (Gy)	49.21 ± 0.66	48.63 ± 0.36	48.82 ±0.48	a, b
D_max_ (Gy)	62.32 ± 2.31	65.77 ± 2.71	68.26 ±3.62	a, b, c
D_min_ (Gy)	46.48 ± 1.19	45.01 ± 1.44	45.50 ±1.18	a, b
D_mean_ (Gy)	55.96 ± 1.10	57.56 ± 1.15	57.62 ±1.45	a, b
PrescriptionIsodose (%)	80.33 ± 2.81	76.15 ± 3.09	73.45 ±3.91	a, b, c
Target coverage (%)	95.83 ±1.10	95.45 ± 0.30	95.66 ±0.69	
CI	1.12 ± 0.07	1.18 ± 0.06	1.25 ± 0.10	a, b, c
HI	1.25 ± 0.05	1.31 ± 0.05	1.36 ± 0.08	a, b, c

a, FSPB vs. FSPB_LS; b, FSPB vs. MC; c, FSPB_LS vs. MC.

The current study established that the prescription dose should cover 100% of the GTV and more than 95% of the PTV. Under this criterion, as depicted in **Figure 1** and **Supplementary Figure 1**, the D_95_ and target coverage derived from the three algorithms were densely clustered around 50 Gy and 95%, respectively, revealing no statistical discrepancy. Concurrently, to satisfy the aforementioned criteria, the MC algorithm required the lowest prescription isodose among the three algorithms, with average and median values of 73.45% ± 3.91% and 73.00%, respectively. For FSPB and FSPB_LS, their mean and median values were 80.33% ± 2.81%, 81.00% and 76.15 ± 3.09, 76.00%, respectively, exhibiting a significant statistical difference among the three algorithms. The lower prescription isodose led to a higher D_max_, D_2_, and D_5_ in the MC plan. The FSPB_LS algorithm showed a statistical difference with the MC only in the D_max_, whereas the FSPB algorithm demonstrated statistical discrepancies with the MC in all three parameters. As shown in **Figure 1**, the third quartiles of the D_max_ for FSPB, FPSB_LS, and MC are 62.50 Gy, 67.35 Gy, and 70.92 Gy, respectively. **Figure 2** compares the dose distribution for representative patients for all three algorithms, with both the MC and FSPB_LS algorithms showing higher dose inside the PTV than FSPB algorithm. The dose parameters, D_98_ and D_min_, were very similar for both the MC and FSPB_LS algorithms, while the FSPB algorithm significantly overestimated these parameters (P<0.05). Additionally, the MC plan demonstrated the highest CI value, with an average of 1.25 ± 0.10, whereas FSPB_LS and FSPB had averages of 1.18 ± 0.06 and 1.12 ± 0.07, respectively, exhibiting significant statistical discrepancies among them. The HI displayed a similar trend with CI among the three algorithms.

**Figure 4:**
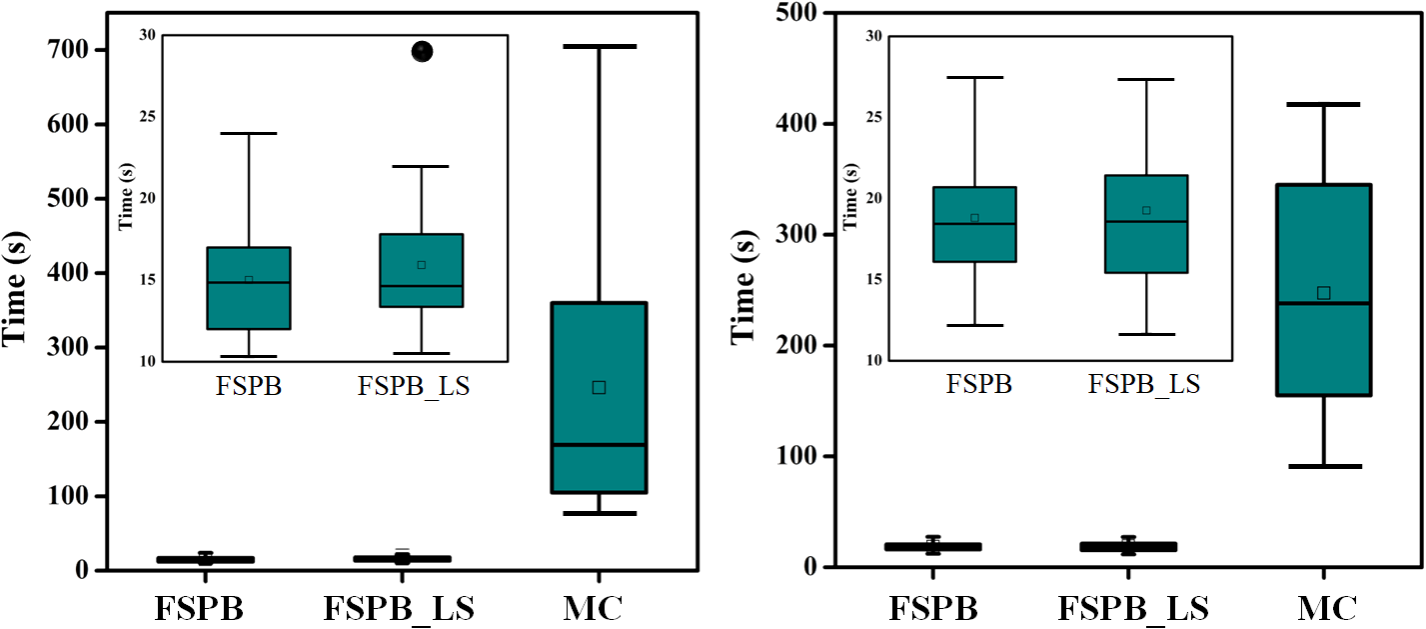
Box plots for computation time of FSPB, PSPB_LS, and MC algorithms for central lung cancer (left panel) and ultra-central lung cancer (right panel). The hollow squares in the plot represent the mean values, while the lines inside the box plots represent the medians. Zoomed FSPB and FSPB_LS were inserted as a subplot.

Moreover, as shown in **Figure 4**, in CLC, the average computation duration of the FSPB algorithm is 15.00 s, with a median duration of 14.96 s. The computation duration ranges from 10.30 s to 24.00 s. In the FSPB_LS algorithm, although lateral scattering is considered, the computation time does not increase significantly. The FSPB_LS algorithm has an average computation duration of 15.93 s, with a median of 14.82 s. Except for one case where the calculation time reached 29 s, the calculation time of other cases ranged between 10.50 s and 21.95 s. Compared to the first two algorithms, the computation duration of the MC algorithm significantly increases. The average computation duration is 246.00 s, with a median of 172.00 s, and the time range is from 77.00 s to 705.00s.

Regarding the OARs of CLC, most of the dosimetric parameters derived from the three algorithms exhibit no statistically significant differences. Simultaneously, the associated violin plots (**Figure 1** and **Supplementary Figure 1**) indicate a similar overall data distribution shape among the three algorithms. The dosimetric data roughly follow the order of MC algorithm being the highest, followed by FSPB_LS, and FSPB being the lowest (**Table 3**). For instance, in the ipsilateral lung, the MC algorithm yields an average D_mean_ of 5.30 Gy ± 2.17 Gy, with a median of 4.72 Gy, a first quartile of 3.62 Gy, and a third quartile of 6.29 Gy. Similarly, the FSPB_LS algorithm yields the following dosimetric parameters: 4.78 Gy ± 2.05 Gy, 4.31 Gy, 3.21 Gy, and 5.81 Gy. The FSPB algorithm further reduces these four dosimetric parameters to 4.53 Gy ± 2.12 Gy, 3.87 Gy, 3.11 Gy, and 5.43 Gy. Similarly, the contralateral lung exhibits the same trend. The DVH of the lungs demonstrated dose differences mainly in the low dose region, while the overlap of the DVH in the high-dose region was seen for three algorithms. For the heart, the dose produced by three algorithms was very low, specifically for V_40_, with almost negligible differences observed among the three algorithms. The D_max_ of the aorta showed significant differences between FSPB and MC algorithms (P<0.05). The dosimetric parameters of the spinal cord, PBT, ribs, and esophagus generated by the MC and PSPB_LS algorithms were slightly greater than those produced by the FSPB algorithm, but this difference was not statistically significant (P>0.05).

**Table 3:** Dose-volumetric parameters of the OARs for CLC.

OARs	DV	FSPB	FSPB_LS	MC
Ipsilateral lung	V_5_ (%)	25.87 ± 12.73	27.48 ± 12.25	30.79 ± 13.43
	V_10_ (%)	11.69 ± 7.55	12.78 ± 7.49	14.15 ± 8.18
	V_20_ (%)	4.59 ± 3.15	5.07 ± 3.13	5.46 ± 3.45
	D_mean_ (Gy)	4.53 ± 2.12	4.78 ± 2.05	5.30 ± 2.17
Contralateral lung	V_5_ (%)	3.57 ± 3.60	4.18 ± 3.55	4.30 ± 4.10
	V_10_ (%)	0.09 ± 0.30	0.17 ± 0.32	0.17 ± 0.40
	V_20_ (%)	0.00 ± 0.00	0.00 ± 0.00	0.00 ± 0.00
	D_mean_ (Gy)	1.08 ± 0.50	1.25 ± 0.68	1.36 ± 0.52
Heart	V_30_ (%)	0.01 ± 0.06	0.06 ± 0.16	0.09 ± 0.22
	V_40_ (%)	0.00 ± 0.00	0.00 ± 0.00	0.00 ± 0.00
	D_mean_ (Gy)	2.04 ± 1.68	2.32 ± 1.82	2.54 ± 1.93
Aorta	D_max_ (Gy)	12.25 ± 7.12	14.02 ± 7.86	15.24 ± 8.19
Spinal cord	D_max_ (Gy)	8.70 ± 8.37	9.82 ± 9.03	10.39 ± 9.46
PBT	D_max_ (Gy)	16.86 ± 9.71	18.77 ± 10.58	19.56 ± 10.70
Esophagus	D_mean_ (Gy)	1.95 ± 1.17	2.14 ± 1.27	2.43 ± 1.34
	D_max_ (Gy)	8.41 ± 4.67	9.55 ± 5.19	10.40 ± 5.70
Ribs	D_max_ (Gy)	25.31 ± 14.52	28.44 ± 15.00	29.68 ± 14.99
	D_1cc_ (Gy)	19.07 ± 12.49	21.65 ± 13.07	22.30 ± 13.42

### Dosimetric comparison of PTV and OARs in ultra-central lung cancer (UCLC)

Among 28 patients suffering from UCLC, the average size of the PTV was 22.45 ± 11.00 cm^3^, varying between 6.0 and 44.2 cm^3^. PTV dose parameters are shown in **Table 4**.

**Table 4:** Dose-volumetric parameters of the PTV for UCLC.

	FSPB	FSPB _LS	MC	P < 0.05
D_2_ (Gy)	61.46 ± 2.52	64.72 ± 2.80	65.42 ± 3.33	a, b
D_5_ (Gy)	60.83 ± 2.45	63.94 ± 2.72	64.40 ± 3.14	a, b
D_95_ (Gy)	50.20 ± 0.11	50.23 ± 0.13	50.27 ± 0.15	
D_98_ (Gy)	48.83 ± 0.27	48.44 ± 0.30	48.46 ± 0.33	a, b
D_max_ (Gy)	62.62 ± 2.54	65.84 ± 2.82	67.51 ± 3.46	a, b
D_min_ (Gy)	45.36 ± 0.97	43.77 ± 1.46	44.14 ± 1.36	a, b
D_mean_ (Gy)	56.33 ± 1.54	58.05 ± 1.42	58.06 ± 1.67	
PrescriptionIsodose (%)	79.97 ± 3.10	76.08 ± 3.24	74.26 ± 3.86	a, b
Target coverage (%)	95.57 ± 0.31	95.47 ± 0.28	95.54 ± 0.31	a, b
CI	1.14 ± 0.07	1.21 ± 0.07	1.27 ± 0.10	a, b, c
HI	1.25 ± 0.05	1.32 ± 0.06	1.35 ± 0.07	a, b

a, FSPB vs. FSPB_LS; b, FSPB vs. MC; c, FSPB_LS vs. MC.

**Figure 5:**
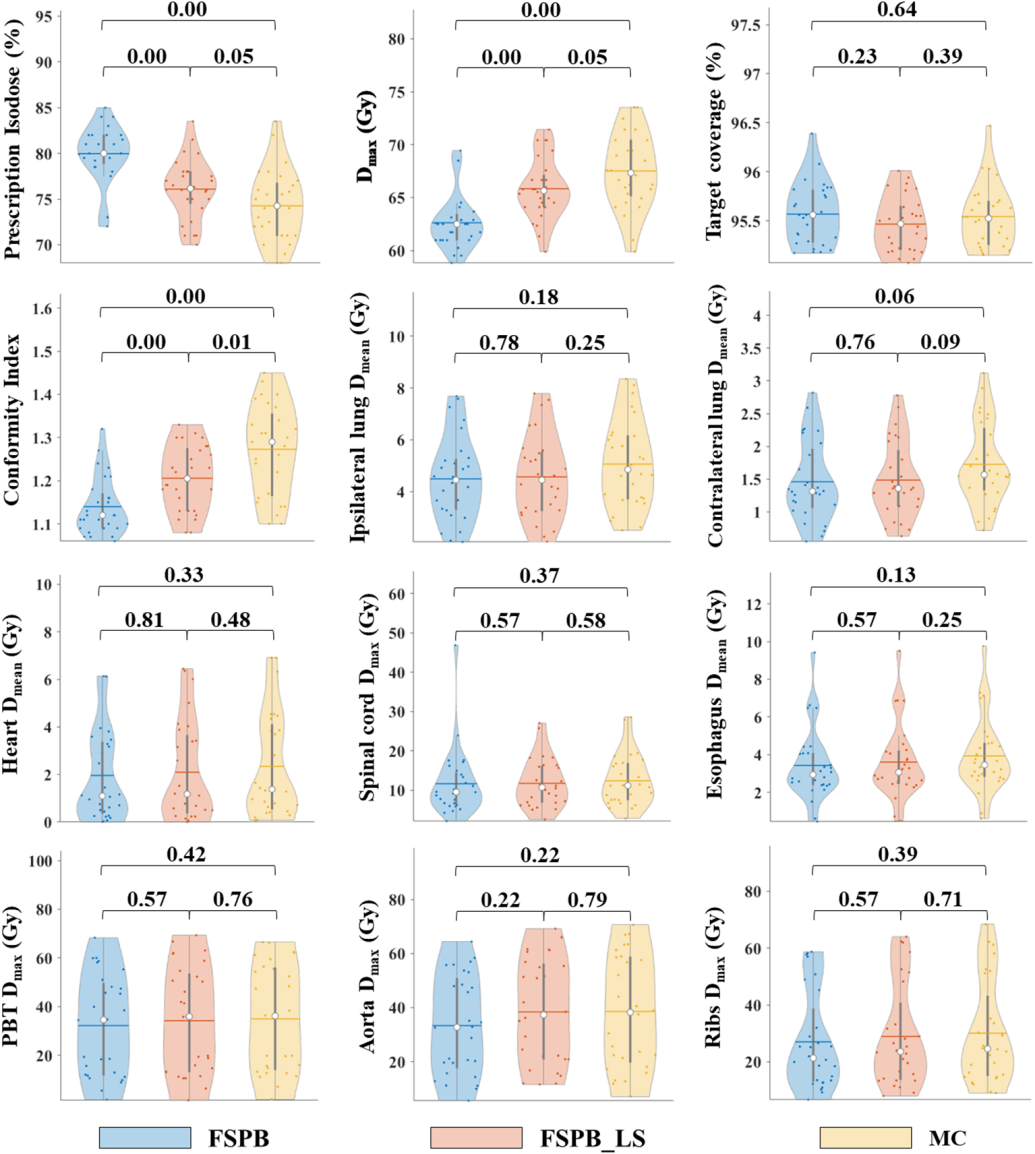
Violin plots of dose parameters for PTV and OARs in UCLC, derived from FSPB, FSPB_LS, and MC algorithms. The horizontal lines within the violin plots represent the mean values, while the hollow circles depict the medians. The P-values are displayed above in the inset.

As the three algorithms follow the mentioned dose normalization, the D_95_ and target coverage of UCLC’s PTV predominantly circulate around 50 Gy and 95% (**Figure 5** and **Supplementary Figure 2**), demonstrating a resemblance with CLC. Nevertheless, in contrast with CLC, no substantial statistical variance was evident between the FSPB_LS and MC algorithms concerning prescription isodose and D_max_. Relative to the MC algorithm, the FSPB_LS algorithm demonstrated a surge of 2.45% in prescription isodose and a decrement of 2.53% in D_max_. Within CLC, these figures amplify to 2.70% and 3.78% correspondingly. These discrepancies are vividly portrayed within the violin plots of CLC and UCLC (refer to **Figures 1**, **5**). Notably, for D_2_, D_5_, D_98_, D_min_ and D_mean_ of PTV, significant discrepancies exist between MC and FSPB algorithms, and between FSPB_LS and FSPB algorithms. Despite this, a considerable difference does not exist between the MC and FSPB_LS algorithms (p< 0.05). Moreover, statistical variances surfaced solely between the FSPB_LS algorithm and the MC algorithm in terms of the CI. The CI yielded by the MC algorithm is 1.27 ± 0.10, whereas the FSPB_LS algorithm generates 1.21 ± 0.07. The HI of the MC, FSPB_LS and FSPB algorithms were found to be 1.35 ± 0.07, 1.32 ± 0.06 and 1.25 ± 0.05, respectively. Among the observed algorithms, the FSPB algorithm produced the lowest HI, with noticeable differences detected between the FSPB_LS and MC algorithms.

As depicted in **Figure 4**, the computation time of FSPB and FSPB_LS is very close, while the computation duration of the MC algorithm is approximately twelve times that of the first two algorithms. Among them, the mean, median, and range of computation time for the FSPB algorithm are 18.81 s, 18.67 s, and 12.20 s to 27.46 s, respectively. For the FSPB_LS algorithm, the mean, median, and range are 19.41 s, 18.96 s, and 11.65 s to 27.35 s, respectively. However, the computation duration of the MC algorithm is significantly different from the first two algorithms in terms of statistical analysis. The mean, median, and range of computation time for the MC algorithm are 247.32 s, 240.00 s, and 91.00 s to 418.00 s, respectively.

For OARs of UCLC such as the lungs, heart, and aorta, the values obtained from the FSPB_LS algorithm are slightly lower than those from the MC algorithm, but slightly higher than those from the FSPB algorithm (**Table 5**). This trend is clearly demonstrated in **Figure 5** and **Supplementary Figure 2**. Additionally, there are no significant statistical differences observed among the three algorithms.

**Table 5:** Dose-volumetric parameters of the OARs for UCLC.

OAR	DV	FSPB	FSPB_LS	MC
Ipsilateral lung	V_5_ (%)	26.65 ±9.43	27.14 ±9.32	30.05 ±9.77
	V_10_ (%)	11.56 ±5.71	11.88 ±5.80	13.34 ±6.15
	V_20_ (%)	4.33 ±2.39	4.53 ±2.53	4.86 ±2.74
	D_mean_ (Gy)	4.49 ±1.58	4.57 ±1.58	5.06 ±1.65
Contralateral lung	V_5_ (%)	6.55 ±5.92	6.48 ±5.61	7.23 ±6.18
	V_10_ (%)	0.23 ±0.45	0.23 ±0.39	0.25 ±0.44
	V_20_ (%)	0.00 ±0.00	0.00 ±0.00	0.00 ±0.00
	D_mean_ (Gy)	1.46 ±0.60	1.49 ±0.57	1.73 ±0.62
Heart	V_30_ (%)	0.05 ±0.16	0.03 ±0.15	0.08 ±0.24
	V_40_ (%)	0.00 ±0.00	0.00 ±0.00	0.00 ±0.00
	D_mean_ (Gy)	1.96 ±1.94	2.09 ±2.06	2.34 ±2.14
Aorta	D_max_ (Gy)	33.31 ±18.01	38.38 ±19.23	38.55 ±20.57
Spinal cord	D_max_ (Gy)	11.61 ±8.40	11.70 ±6.03	12.36 ±6.35
PBT	D_max_ (Gy)	32.19 ±20.43	34.19 ±21.62	34.95 ±21.53
Esophagus	D_mean_ (Gy)	3.43 ±1.88	3.61 ±1.93	3.93 ±1.94
	D_max_ (Gy)	17.92 ±12.69	19.32 ±13.28	20.02 ±13.16
Ribs	D_max_ (Gy)	27.00 ±16.81	28.91 ±17.70	30.04 ±17.70
	D_1cc_ (Gy)	20.97 ±15.20	22.19 ±14.88	22.79 ±15.21

**Figure 6:**
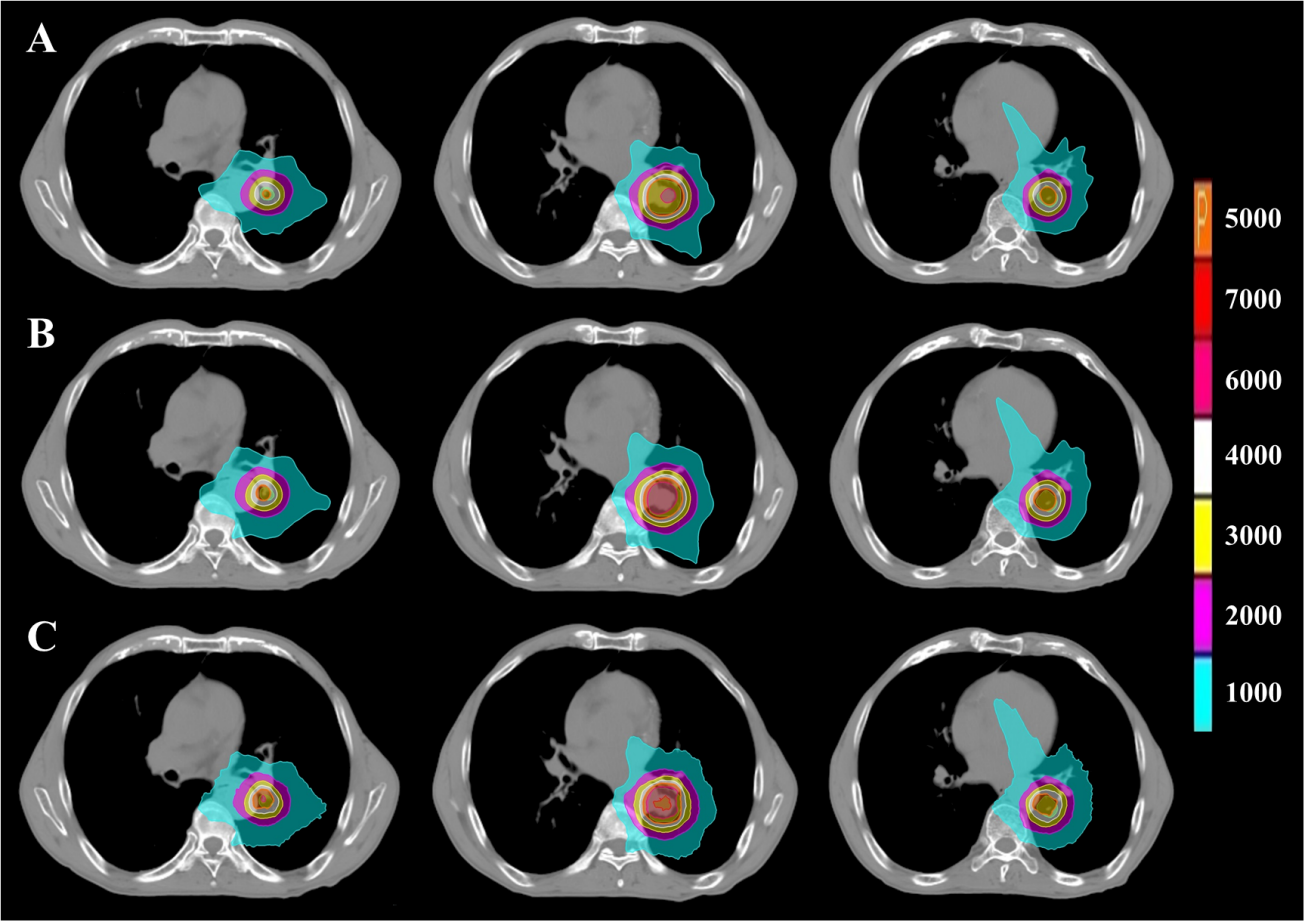
PTV dose distribution of representative patient with UCLC based on FSPB, FSPB_LS and MC algorithms. The images from top to bottom illustrate **(A)** FSPB, **(B)** FSPB_LS, and **(C)** MC algorithms respectively. The images from left to right demonstrate the dose distribution of top, middle, and bottom layers of PTV along superior-inferior (S-I) direction. P represents the Prescription Dose.

**Figure 7:**
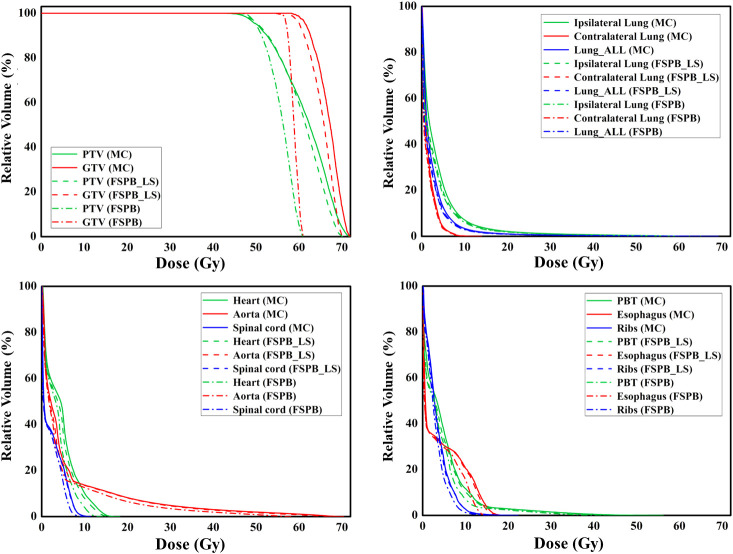
The dose-volume histograms (DVH) of PTV, GTV, and OARs planned through FSPB, FSPB_LS, and MC algorithms for the representative patient with UCLC.

**Figure 6** displays representative patient dose distributions while **Figure 7** showcases the DVHs. In PTV, the FSPB_LS algorithm exhibited strong consistency with the MC algorithm, whereas in the FSPB algorithm, the high-dose region within the PTV was significantly underestimated. **Figure 6** records that the volume of the high-dose region produced by the FSPB algorithm is notably lower than those generated by the other two algorithms, in agreement with DVH. In connection with GTV, these dose parameters indicated the same trend as that observed in PTV. The lungs DVH shapes of the three algorithms were almost identical, with only slight variations observed in the low-dose region of the ipsilateral lung. Within representative patients, PTV overlapped with the aorta, and the maximum dose that the FSPB, FSPB_LS, and MC algorithms generated for the aorta were 58.40Gy, 69.25Gy, and 70.68Gy, correspondingly. For the heart, the three algorithms indicate lower D_mean_. The DVH of the heart indicated that the FSPB_LS algorithm demonstrated a high degree of accuracy, on par with the MC algorithm, while the FSPB algorithm greatly underestimated the dose to the heart. The same tendency was also observed for the esophagus and PBT. In relation to the spinal cord and ribs, the difference between these algorithms was not notable in DVH.

## Discussions

With respect to CLC, the FSPB_LS algorithm is able to improve the ability of the FSPB algorithm to handle non-uniform regions to a certain extent. Only in a few dosimetric parameters of the PTV does the FSPB_LS algorithm exhibit differences with MC. This is mainly because the beam passes through lengthy pathways of low-density lung tissue before it arrives at the CLC. The MC algorithm sufficiently considers the dose deposited in low-density media by correcting for low-density photon scattering and electron equilibrium in heterogeneous geometric structures. Precise modeling of the secondary electron disequilibrium at the tumor-lung tissue interface leads to increased penumbra of the beam which results in poorer consistency and uniformity of the PTV, compared to the FSPB_LS plan (28). To comply with the RTOG 0813 protocol (29), a lower prescription isodose for PTV was determined. The prescription isodose in the MC algorithm is reduced by 6.9% when compared to FSPB, and only by 2.7% when compared to FSPB_LS. The significant reduction in prescription isodose for the FSPB algorithm indicates the lack of accuracy of this non-uniform region dose, fundamentally because the FSPB algorithm carries out density correction only for the contribution of primary photon and does not reproduce the physics effects that cause it. Conversely, the FSPB_LS algorithm executes lateral scattering correction, which is believed to have significantly contributed to the dose consistency with MC.

Insubstantial differences in dose were shown among the three algorithms for OARs of the CLC. This study’s results align with previous studies that compared dosimetric parameters among CK algorithms (30). Only differences in dosimetric parameters for V_10_ in the contralateral lung were observed between FSPB and FSPB_LS, whereas statistically significant differences were observed between FSPB and MC in D_mean_ of the contralateral lung and D_max_ of the aorta. The FSPB algorithm underestimates lung dose, which could lead to incorrect judgement by radiation oncologists, thus increasing the PTV prescription dose and causing normal tissue intolerance (31). Additionally, underestimation of aorta’s D_max_ by the FSPB algorithm could result in excessive radiation exposure, causing severe side effects of radiation (32). However, there were no statistically significant dosimetric differences observed for all other OARs between FSPB_LS and MC. When comparing the two algorithms, the dose deviation for PTV is between 0.10% and 3.65%, whereas the range of dose deviation for OARs is from 2.79% to 11.93%. Currently, the American Association of Physicists in Medicine (AAPM) recommends that the overall dose accuracy of radiation therapy is at 5%. The dose calculation should be maintained within 3% (7). Although the FSPB_LS algorithm can significantly improve the accuracy of the FSPB algorithm, there still exists a gap compared to the MC algorithm. Therefore, caution should be exercised while using the FSPB_LS algorithm for the final dose calculation in the CLC to ensure the safe implementation of SBRT.

In general, the majority of dosimetric parameters in the violin plot depicting PTV and OARs exhibit a consistent trend: MC > FSPB_LS > FSPB. The FSPB algorithm demonstrates substantial differences in PTV dose parameters from the MC algorithm for UCLC. In comparison to CLC, the PSPB_LS algorithm exhibits greater consistency with the MC algorithm for UCLC, with only statistical differences noted for CI. The improved consistency may be attributed to UCLC overlapping with soft tissues and showing smaller density changes, leading to a reduced electron secondary imbalance. In this scenario, the FSPB_LS algorithm can perform lateral scaling correction to enhance dose calculation accuracy. However, upon implementing a unified normalization standard, the CI associated with the MC algorithm significantly surpasses that of the other two algorithms. According to the formula (1) in METHODS AND MATERIALS, this discrepancy indicates an underestimation of the prescribed dose’s coverage range by the other two algorithms, potentially leading to heightened delivered doses and radiation-induced side effects (33).

Diametric parameters of OARs showed no statistically significant differences in patients with UCLC. However, algorithm and prescription settings for UCLC must be approached with caution given its overlap with critical structures such as PBT, aortas, and esophagus. After SBRT treatment in UCLC patients, a portion of patients were found to develop acute esophagitis while a few experienced tracheoesophageal fistula (20, 34). Additionally, another study found that 14% of patients died from bronchopulmonary hemorrhages after receiving 60 Gy/12f for UCLC and patients with main bronchial D_mean_ BED_3_ of 91 Gy significantly increased the likelihood of ≥ grade 3 toxicity (35). Van der Voort van Zyp et al. (36) on CK treatment of NSCLC, it was suggested that the prescription dose should be set separately according to tumor size for the conversion from equivalent path length (EPL) algorithm to MC.

The timing of dose calculations is a consideration for radiation physicists. The FSPB_LS algorithm greatly reduces the computation duration of the MC algorithm. In this study, the scanning layer thickness of the cases we used was 5mm. However, in actuality, cases treated with CK generally employ thin-layer scanning, which further exacerbates the time difference between the MC algorithm and other algorithms. Although the MC algorithm requires significant computation time, through a series of data comparisons and analysis in this paper, it is found that the FSPB_LS algorithm still cannot perfectly replicate the precise dose delivery in the lungs achieved by the MC algorithm. Considering the numerous OARs surrounding CLC and UCLC, we believe that the use of the FSPB_LS algorithm should be cautious. And the use of inappropriate algorithms may lead to misjudgments by radiation oncologists. In clinical treatment, estimating tumor control and radiation-induced side effects through dose distribution is highly important. Although the FSPB_LS and FSPB algorithms can generate dose distributions in a short amount of time, they suffer from dose calculation discrepancies compared to MC, greatly outweighing their advantages in terms of time cost. If only the FSPB_LS algorithm can be used for dose calculation, we recommend setting the dose calculation resolution to “High”. Additionally, before plan execution, institutions should establish more rigorous dose verification standards.

It should be noted that this study employed the M6 with TPS version Precision 1.1.1.1. Considering the maturity of the market for CK, differences among accelerators can be negligible. Hence, institutions using the same model of CK can refer to the results of this study. Furthermore, based on the location of lesions, lung cancer can be categorized into peripheral, central, and super-central. This study only focuses on CLC and UCLC and does not explore peripheral lung cancer. Given that peripheral lung cancer is surrounded by low-density lung tissue, which is significantly different from CLC and UCLC, the conclusions of this study may not be applicable to peripheral cases.

The length of the low-density lung tissue pathway traversed by the beam is an essential factor that influences dose calculation accuracy. We recommend utilizing the prone position for patients with tumors located proximately to the spinal region during positioning. During planning optimization, optimizing dose distribution accuracy can be achieved by setting avoidance regions with reduced lung penetration. While not yet published, we believe that this approach can enhance dose calculation accuracy.

Here are some limitations and prospects of this research. As a retrospective study, patients were not randomized, resulting in potential selection bias. In addition, the lack of attention to the influence of CT scan layer thickness on dose calculation accuracy. All patients in this study had been historically treated with SBRT on a True Beam accelerator, utilizing a 3 mm scan layer thickness. However, CK planning in Precision TPS typically utilizes thin-layer scanning (1-1.5 mm). The effect of varying scan layer thicknesses on the accuracy of the three dose algorithms remains indeterminate. Moreover, in previous treatments, patients had their arms raised. However, during CyberKnife treatment, to avoid collisions during robotic arm movement, patients typically place their arms on both sides of the body. These limiting factors could potentially impact dose calculations.

In the future, we can evaluate the impact of different algorithms on patient clinical outcomes through long-term multi-institutional follow-up observations. Furthermore, utilizing the built-in biological assessment module in TPS, evaluating the impact of different algorithms on tumor control probability (TCP) and normal tissue complications probability (NTCP) in terms of dose differences is considered a future research direction (33, 37, 38). This study can assist radiation oncologists in prospectively understanding the impact of these three algorithms on patient prognosis. Unfortunately, Precision 1.1.1.1 used in this study does not have a similar module. We hope that with future updates and iterations of algorithms, we can conduct research addressing this issue. Currently, we have only compared the differences among these three algorithms in CLC and UCLC. We plan to conduct similar studies concerning peripheral lung cancer. Considering the proximity of peripheral lung cancer to surrounding OARs, this may lead to differences that are completely distinct from the results of this study.

## Conclusion

This study compared three different CK dose algorithms based on CLC and UCLC cases. The results showed that most dose parameters of PTV and OARs demonstrated the trend of MC > FSPB_LS > FSPB. The FSPB_LS algorithm significantly improved the calculation accuracy of the traditional FSPB algorithm and exhibited dose distributions similar to MC within the PTV. However, for CLC, there were significant differences between the FSPB_LS algorithm and the MC algorithm in terms of OARs. Although these differences reduced in UCLC, the OARs in UCLC were highly sensitive to dose variations. In conclusion, we recommend using the MC algorithm for dose calculation in CLC and UCLC. The findings of this study provide important guidance for algorithm selection in MLC-based CK planning, helping radiation oncologists gain intuitive insights into the differences among various dose algorithms in CLC and UCLC, and offering beneficial references for clinical algorithm choices.

## Data availability statement

The original contributions presented in the study are included in the article/**Supplementary Material**. Further inquiries can be directed to the corresponding authors.

## Ethics statement

The retrospective research of the medical records was approved by Institutional Review Board of the Shandong Cancer Hospital for this analysis.

## Author contributions

QQ and YY conceived the concept and designed the experiment. XCG and MY performed the experiments. XCG, MY, and QQ analyzed the data. XCG, MY, and QQ prepared the manuscript. TXL, THL, and XYG contributed to the improvement of the manuscript. All authors contributed to the article and approved the submitted version.

## References

[1] Biswas T Holland B Rosenman J Podder T. SU-E-T-422: lung SBRT using cyberknife: technique and treatment outcome. Med Phys (2012) 39(6Part16):3801–02. doi: 10.1118/1.4735511 28517207

[2] Serra M Ametrano G Borzillo V Quarto M Muto M Di Franco R. Dosimetric comparison among cyberknife, helical tomotherapy and VMAT for hypofractionated treatment in localized prostate cancer. Medicine (2020) 99(50):e23574. doi: 10.1097/MD.0000000000023574 33327317 PMC7738085

[3] Yu S Xu H Sinclair A Zhang X Langner U. Mak K Dosimetric and planning efficiency comparison for lung SBRT: CyberKnife vs VMAT vs knowledge-based VMAT. Med Dosim (2020) 45(4):346–51. doi: 10.1016/j.meddos.2020.04.004 32532613

[4] Kaul D Badakhshi H Gevaert T Pasemann D Budach V Tulaesca C. Dosimetric comparison of different treatment modalities for stereotactic radiosurgery of meningioma. Acta Neurochir (2015) 157:559–64. doi: 10.1007/s00701-014-2272-9 25413163

[5] Diamant A Heng VJ Chatterjee A Faria S Bahig H Filion E. Comparing local control and distant metastasis in NSCLC patients between CyberKnife and conventional SBRT. Radiother Oncol (2020) 144:201–08. doi: 10.1016/j.radonc.2020.01.017 32044418

[6] Zvolanek K Ma R Zhou C Liang X Wang S Verma V. Still equivalent for dose calculation in the Monte Carlo era? A comparison of free breathing and average intensity projection CT datasets for lung SBRT using three generations of dose calculation algorithms. Med Phys (2017) 44(5):1939–47. doi: 10.1002/mp.12193 28273341

[7] Papanikolaou N Battista J Boyer A Kappas C Klein E Mackie T. AAPM Report No. 85: tissue inhomogeneity corrections for megavoltage photon beams. Rep Task Group (2004) 65:1–142.

[8] Bourland J Chaney E. A finite-size pencil beam model for photon dose calculations in three dimensions. Med Phys (1992) 19(6):1401–12. doi: 10.1118/1.596772 1461202

[9] Jeleń U Söhn M Alber M. A finite size pencil beam for IMRT dose optimization. Phys Med Biol (2005) 50(8):1747. doi: 10.1088/0031-9155/50/8/009 15815094

[10] Jeleń U Alber M. A finite size pencil beam algorithm for IMRT dose optimization: density corrections. Phys Med Biol (2007) 52(3):617. doi: 10.1088/0031-9155/52/3/006 17228109

[11] Deng J Ma CM Hai J Nath R. Commissioning 6 MV photon beams of a stereotactic radiosurgery system for Monte Carlo treatment planning. Med Phys (2003) 30(12):3124–34. doi: 10.1118/1.1624753 14713079

[12] Deng J Guerrero T Ma C Nath R. Modelling 6 MV photon beams of a stereotactic radiosurgery system for Monte Carlo treatment planning. Phys Med Biol (2004) 49(9):1689. doi: 10.1088/0031-9155/49/9/007 15152924

[13] Li J Zhang X Pan Y Zhuang H Yang R. Comparison of ray tracing and monte carlo calculation algorithms for spine lesions treated with cyberKnife. Front Oncol (2022) 12:898175. doi: 10.3389/fonc.2022.898175 35600341 PMC9116717

[14] Kawata K Kamomae T Oguchi H Kawabata F Okudaira K Kawamura M. Evaluation of newly implemented dose calculation algorithms for multileaf collimator-based CyberKnife tumor-tracking radiotherapy. Med Phys (2020) 47(3):1391–403. doi: 10.1002/mp.14013 31913508

[15] Wang B Dong Y Yu X Li F Wang J Chen H. Safety and efficacy of stereotactic ablative radiotherapy for ultra-central lung cancer. Front Oncol (2022) 12:868844. doi: 10.3389/fonc.2022.868844 35600391 PMC9118536

[16] Tan H Nusrat H Li G Poon I Tsao M Ung Y. Safety and efficacy of stereotactic body radiotherapy for ultra-central thoracic tumors. Int J Radiat Oncol Biol Phys (2021) 111(3):e453–e54. doi: 10.1016/j.ijrobp.2021.07.1275 38621607

[17] Timmerman R Mcgarry R Yiannoutsos C Papiez L Tudor K Deluca J. Excessive toxicity when treating central tumors in a phase II study of stereotactic body radiation therapy for medically inoperable early-stage lung cancer. J Clin Oncol (2006) 24(30):4833–39. doi: 10.1200/JCO.2006.07.5937 17050868

[18] Chaudhuri AA Tang C Binkley MS Jin M Wynne JF Von Eyben R. Stereotactic ablative radiotherapy (SABR) for treatment of central and ultra-central lung tumors. Lung Cancer (2015) 89(1):50–6. doi: 10.1016/j.lungcan.2015.04.014 25997421

[19] Unger K Ju A Oermann E Suy S Yu X Vahdat S. CyberKnife for hilar lung tumors: report of clinical response and toxicity. J Hematol Oncol (2010) 3(1):1–7. doi: 10.1186/1756-8722-3-39 20969774 PMC2987864

[20] Wang C Rimner A Gelblum DY Dick-Godfrey R Mcknight D Torres D. Analysis of pneumonitis and esophageal injury after stereotactic body radiation therapy for ultra-central lung tumors. Lung Cancer (2020) 147:45–8. doi: 10.1016/j.lungcan.2020.07.009 PMC7484437 32663723

[21] Nguyen KNB Hause DJ Novak J Monjazeb AM Daly ME. Tumor control and toxicity after SBRT for ultracentral, central, and paramediastinal lung tumors. Pract Radiat Oncol (2019) 9(2):e196–202. doi: 10.1016/j.prro.2018.11.005 PMC6402805 30496842

[22] Chang JY Bezjak A Mornex F. Committee I a R T Stereotactic ablative radiotherapy for centrally located early stage non–small-cell lung cancer: what we have learned. J Thorac Oncol (2015) 10(4):577–85. doi: 10.1097/JTO.0000000000000453 25514807

[23] Underberg RWM Lagerwaard FJ Slotman BJ Cuijpers JP Senan S. Use of maximum intensity projections (MIP) for target volume generation in 4DCT scans for lung cancer. Int J Radiat Oncol Biol Phys (2005) 63(1):253–60. doi: 10.1016/j.ijrobp.2005.05.045 16111596

[24] Shirai K Nishiyama K Katsuda T Teshima T Ueda Y Miyazaki M. Phantom and clinical study of differences in cone beam computed tomographic registration when aligned to maximum and average intensity projection. Int J Radiat Oncol Biol Phys (2014) 88(1):189–94. doi: 10.1016/j.ijrobp.2013.09.031 24331666

[25] Group. R T O. A phase II trial of stereotactic body radiation therapy in the treatment of patient with medically inoperable stage I/II non-small cell lung cancer. RTOG 0236 (2009) 1–39.

[26] Wu VW Tam KW Tong SM. Evaluation of the influence of tumor location and size on the difference of dose calculation between Ray Tracing algorithm and Fast Monte Carlo algorithm in stereotactic body radiotherapy of non-small cell lung cancer using CyberKnife. J Appl Clin Med Phys (2013) 14(5):68–78. doi: 10.1120/jacmp.v14i5.4280 24036860 PMC5714561

[27] Heidorn SC Kilby W Furweger C. Novel Monte Carlo dose calculation algorithm for robotic radiosurgery with multi leaf collimator: Dosimetric evaluation. Phys Med (2018) 55:25–32. doi: 10.1016/j.ejmp.2018.10.011 30471816

[28] Wilcox EE Daskalov GM Lincoln H Shumway RC Kaplan BM Colasanto JM. Comparison of planned dose distributions calculated by Monte Carlo and Ray-Trace algorithms for the treatment of lung tumors with cyberknife: a preliminary study in 33 patients. Int J Radiat Oncol Biol Phys (2010) 77(1):277–84. doi: 10.1016/j.ijrobp.2009.08.001 20004530

[29] Bezjak A Bradley J Gaspar L Robert D Papiez L Gore E. Seamless phase I/II study of stereotactic lung radiotherapy (SBRT) for early stage, centrally located, non-small cell lung cancer (NSCLC) in medically inoperable patients. RTOG (2012) 813:1–75.

[30] Dona KNUG Shang C Leventouri T. Dosimetric Comparison of treatment plans computed with finite size pencil beam and monte carlo algorithms using the incise™ multileaf collimator-equipped cyberknife® system. J Med Phys (2020) 45(1):7. doi: 10.4103/jmp.JMP_64_19 32355430 PMC7185708

[31] Okoye CC Patel RB Hasan S Podder T Khouri A Fabien J. Comparison of ray tracing and Monte Carlo calculation algorithms for thoracic spine lesions treated with CyberKnife-based stereotactic body radiation therapy. Technol Cancer Res Treat (2016) 15(1):196–202. doi: 10.1177/1533034614568026 25633137

[32] Evans JD Gomez DR Amini A Rebueno N Allen PK Martel MK. Aortic dose constraints when reirradiating thoracic tumors. Radiother Oncol (2013) 106(3):327–32. doi: 10.1016/j.radonc.2013.02.002 PMC3921976 23453540

[33] Liang X Penagaricano J Zheng D Morrill S Zhang X Corry P. Radiobiological impact of dose calculation algorithms on biologically optimized IMRT lung stereotactic body radiation therapy plans. Radiother Oncol (2016) 11(1):1–11. doi: 10.1186/s13014-015-0578-2 PMC4724090 26800883

[34] Sodji QH Ko R Von Eyben R Owen SG Capaldi DPI Bush K. Acute and late esophageal toxicity after SABR to thoracic tumors near or abutting the esophagus. Int J Radiat Oncol Biol Phys (2022) 112(5):1144–53. doi: 10.1016/j.ijrobp.2021.12.008 34942312

[35] Lodeweges JE Van Rossum PSN Bartels M Van Lindert ASR Pomp J Peters M. Ultra-central lung tumors: safety and efficacy of protracted stereotactic body radiotherapy. Acta Oncol (2021) 60(8):1061–68. doi: 10.1080/0284186X.2021.1942545 34191670

[36] Van Der Voort Van Zyp NC Hoogeman MS Van De Water S Levendag PC van der Holt B Heijmen BJ. Clinical introduction of Monte Carlo treatment planning: a different prescription dose for non-small cell lung cancer according to tumor location and size. Radiother Oncol (2010) 96(1):55–60. doi: 10.1016/j.radonc.2010.04.009 20430461

[37] Duan Y Cao H Wu B Wu Y Liu D Zhou L. Dosimetric comparison, treatment efficiency estimation, and biological evaluation of popular stereotactic radiosurgery options in treating single small brain metastasis. Front Oncol (2021) 11:716152. doi: 10.3389/fonc.2021.716152 34540686 PMC8447903

[38] Nielsen TB Wieslander E Fogliata A Nielsen M Hansen O Brink C. Influence of dose calculation algorithms on the predicted dose distributions and NTCP values for NSCLC patients. Med Phys (2011) 38(5):2412–18. doi: 10.1118/1.3575418 21776775

