# Supplementary material for: Comparative analysis of dose calculation algorithms for CyberKnife-based stereotactic radiotherapy in lung cancer
Source: Front Oncol. 2023 Oct 2;13:1215976. doi: 10.3389/fonc.2023.1215976 (PMC10577380; doi:10.3389/fonc.2023.1215976)
Supplement: Supplementary file 1 [file DataSheet_1.docx]

**Comparative Analysis of Dose Calculation Algorithms for CyberKnife-based Stereotactic Radiotherapy in Lung Cancer**

*Xuanchu Ge^1,#^, Mingshan Yang^2,#^, Tengxiang Li^1^,Tonghai Liu^1^, Xiangyu Gao^3^,Qingtao Qiu*^,1^, and Yong Yin^*,1^*

*^1^Department of Radiation Oncology and Physics, Shandong Cancer Hospital and Institute, Shandong First Medical University and Shandong Academy of Medical Sciences, Jinan, China, ^2^Department of Urology, Shandong Cancer Hospital and Institute, Shandong First Medical University and Shandong Academy of Medical Sciences, Jinan, China, ^3^Department of Radiation Oncology, Qilu Hospital of Shandong University, Cheeloo College of Medicine, Shandong University, Jinan, China.*

*Correspondence to: Qingtao Qiu; Yong Yin. Department of Radiation Oncology and Physics, Shandong Cancer Hospital and Institute, Shandong First Medical University and Shandong Academy of Medical Sciences, Jinan, China. Email: qiuqingt@126.com; yinyongsd@126.com.*


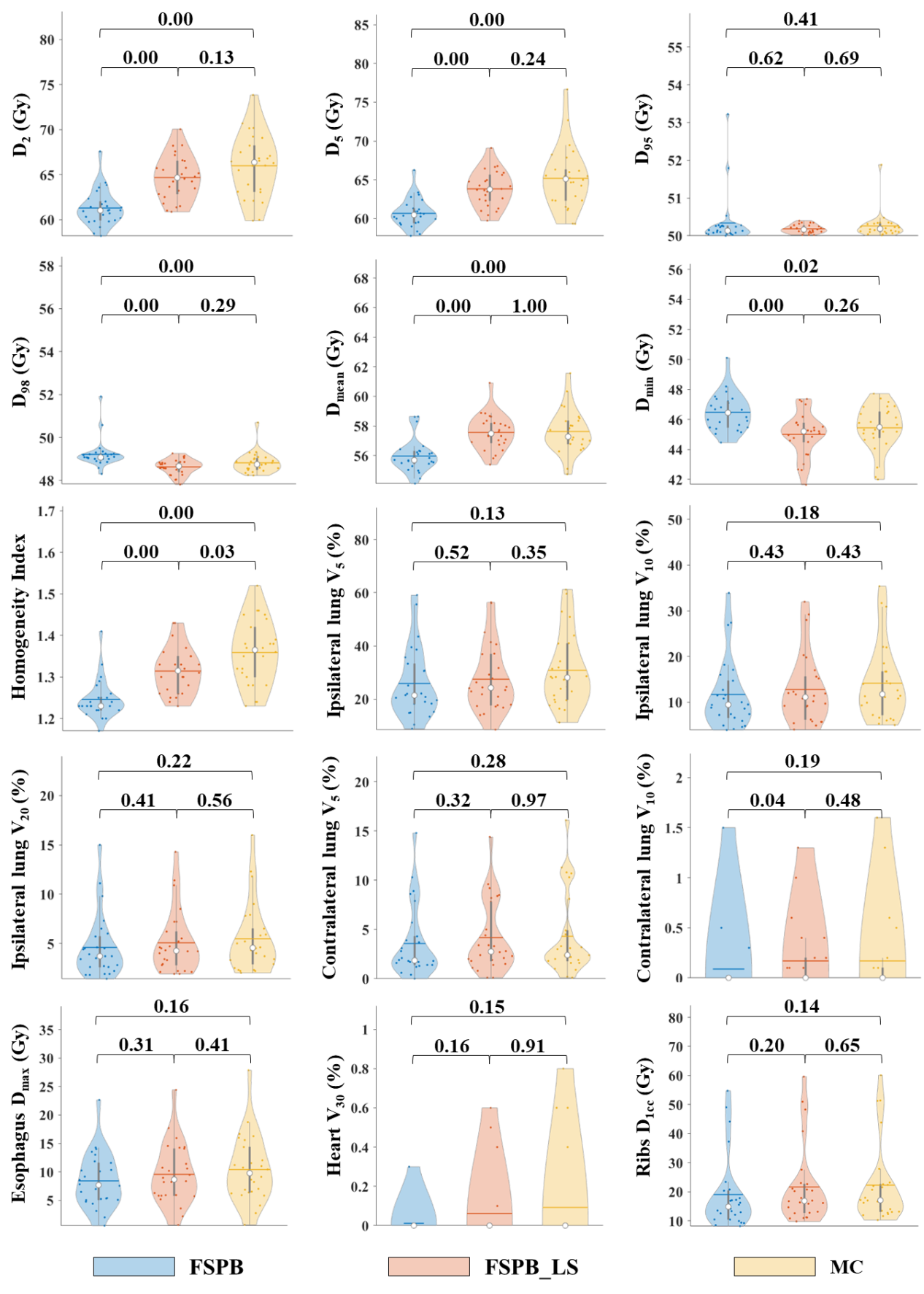


Figure S1. Violin plots of partly dose parameters for PTV and OARs in CLC, derived from FSPB, FSPB_LS, and MC algorithms. The horizontal lines within the violin plots represent the mean values, while the hollow circles depict the medians. The P-values are displayed above in the inset.


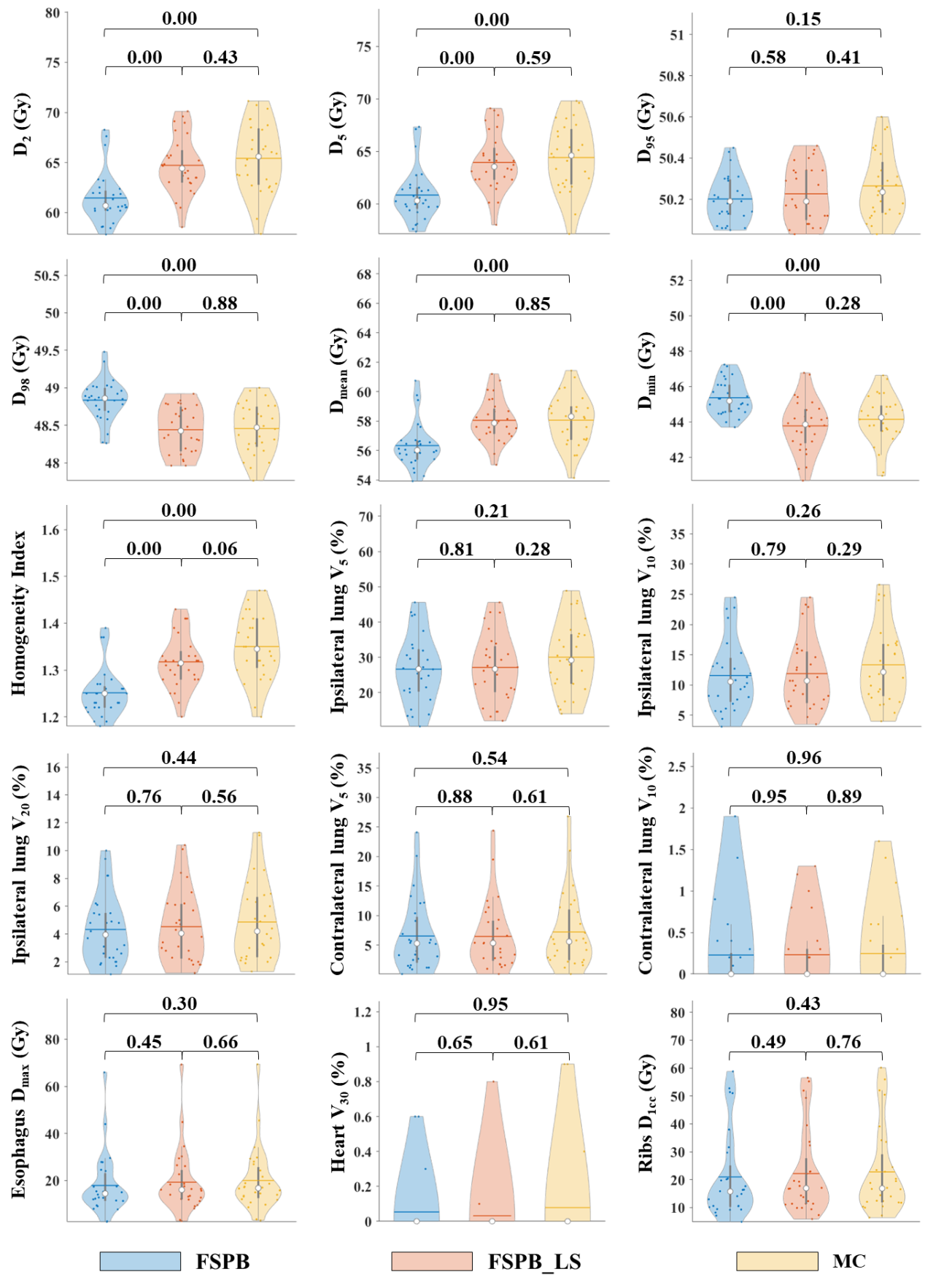


Figure S2. Violin plots of partly dose parameters for PTV and OARs in UCLC, derived from FSPB, FSPB_LS, and MC algorithms. The horizontal lines within the violin plots represent the mean values, while the hollow circles depict the medians. The P-values are displayed above in the inset.
